# Antimicrobial Activities of Cysteine-rich Peptides Specific to Bacteriocytes of the Pea Aphid *Acyrthosiphon pisum*

**DOI:** 10.1264/jsme2.ME18148

**Published:** 2019-03-21

**Authors:** Nahoko Uchi, Mitsutaka Fukudome, Narumi Nozaki, Miyuzu Suzuki, Ken-ichi Osuki, Shuji Shigenobu, Toshiki Uchiumi

**Affiliations:** 1 Graduate School of Science and Engineering, Kagoshima University 1–21–35 Korimoto, Kagoshima, Kagoshima 890–0065 Japan; 2 National Institute for Basic Biology 38 Nishigonaka, Myodaiji, Okazaki, Aichi 444–8585 Japan

**Keywords:** symbiosis, aphid, *Buchnera*, cysteine-rich peptide, bacteriocyte

## Abstract

Aphids have a mutualistic relationship with the bacterial endosymbiont *Buchnera aphidicola*. We previously reported seven cysteine-rich peptides in the pea aphid *Acyrthosiphon pisum* and named them Bacteriocyte-specific Cysteine-Rich (BCR) peptides; these peptides are exclusively expressed in bacteriocytes, special aphid cells that harbor symbionts. Similar symbiotic organ-specific cysteine-rich peptides identified in the root nodules of leguminous plants are named Nodule-specific Cysteine-Rich (NCR) peptides. NCR peptides target rhizobia in the nodules and are essential for symbiotic nitrogen fixation. A BacA (membrane protein) mutant of *Sinorhizobium* is sensitive to NCR peptides and is unable to establish symbiosis. Based on the structural and expressional similarities between BCR peptides and NCR peptides, we hypothesized that aphid BCR peptides exhibit antimicrobial activity, similar to some NCR peptides. We herein synthesized BCR peptides and investigated their antimicrobial activities and effects on the bacterial membrane of *Escherichia coli*. The peptides BCR1, BCR3, BCR5, and BCR8 exhibited antimicrobial activities with increased membrane permeability. An *sbmA* mutant of *E. coli*, a homolog of *bacA* of *S. meliloti*, was more sensitive to BCR peptides than the wild type. Our results suggest that BCR peptides have properties that may be required to control the endosymbiont, similar to NCR peptides in legumes.

Endosymbiosis is often essential for the survival of hosts and symbionts. A well-studied example is the mutual interdependence between aphids and *Buchnera* (30). Aphids harbor an endosymbiotic γ-proteobacterium, *Buchnera aphidicola*, within specialized cells called bacteriocytes (27). *Buchnera* provides the host with nutrients, such as essential amino acids, that aphids cannot synthesize and that are deficient in plant phloem sap, aphids’ sole dietary component (13, 29). The relationship between aphids and *Buchnera* is syntrophic and obligate. *Buchnera* cells are vertically transmitted through host generations by transvariole transfer: they are exocytosed from the maternal bacteriocyte, temporarily released into the extracellular space, and endocytosed by the posterior syncytial cytoplasm of the blastula during early embryogenesis (stage 7) (4, 5, 17, 26). This symbiotic relationship is estimated to have been established 200–250 Myr ago. This long-term endosymbiotic relationship has shaped the characteristic streamlined genome, from which *Buchnera* has lost many genes, including those involved in the biosynthesis of lipopolysaccharides and phospholipids, gene regulation, and defense responses, and has, thus, lost the ability to survive outside of host bacteriocytes (2, 29).

Seven cysteine-rich peptides (CRPs) that are exclusively expressed in the bacteriocytes of the pea aphid *Acyrthosiphon pisum* have been identified and designated as “BCRs” (Bacteriocyte-specific Cysteine-Rich [BCR] peptides) (31). Each BCR peptide consists of a secretion signal peptide and mature peptide (44–84 amino acids) with 6 or 8 cysteine residues (31). Their expression was initially detected in stage 7 embryos, with *Buchnera* cells being transported from maternal bacteriocytes to the embryonic syncytium, and bacteriocyte-specific expression is then maintained throughout the rest of the aphid’s life. Although this expression pattern suggests the importance of BCRs in the symbiosis with *Buchnera*, their physiological activities and functions in symbiosis currently remain unknown.

CRPs in symbiosis organs are found in other symbioses, such as that between legumes and nitrogen-fixing *α-Proteobacteria* called rhizobia (22), actinorhizal plants and nitrogen-fixing *Frankia* (6), and bean bugs and *β-Proteobacteria* of the genus *Burkholderia* (10). In the legume symbiosis, the host plant forms a specific organ, the root nodule, in which rhizobia live. Rhizobia penetrate the nodule cells and differentiate into bacteroids, the symbiotic form. By metabolic adaptation, bacteroids gain the ability to fix nitrogen (22, 33). In nodules formed on the roots of legumes, such as *Medicago*, *Pisum*, and *Trifolium*, bacteroids show cell elongation, genome amplification, cell membrane modifications, and the loss of reproductive activity (23). This terminal differentiation is mediated by nodule-specific cysteine-rich (NCR) peptides that are produced by the host plants (34, 35).

*Medicago truncatula* produces more than 600 NCR peptides in infected nodule cells only (28). NCR peptides are structurally similar to defensins, *i.e*., they have signal peptides and mature peptides that conserve 4 or 6 cysteine residues (22). Some NCR peptides exhibit antimicrobial activity (34). Synthesized NCR peptides have the ability to induce cell elongation, polyploidization, and cell membrane modifications in *Sinorhizobium meliloti* cultured *in vitro* (34). Signal peptides are cleaved by signal peptidase, and mature NCR peptides are delivered to the microsymbionts inside host plant cells. The *DNF1* gene of *M. truncatula* encodes a subunit of a nodule-specific signal peptidase; *dnf1* mutants cannot establish effective symbiosis (32, 35). In *dnf1* mutant nodules, rhizobia remain undifferentiated, and NCR peptides localize within the endoplasmic reticulum, and, thus, are not delivered to bacteroids. These findings strongly support the view that NCR peptides are essential for effective symbiosis (34, 35).

To survive exposure to NCR peptides, *S. meliloti* requires BacA (14). The *S. meliloti bacA* mutant is hypersensitive to NCR peptides: when *S. meliloti bacA* mutant cells are released into nodule cells, they are rapidly killed (14). However, they may survive in the nodule cells of the *dnf1* mutant because NCR peptides are not transported to these cells. These findings show that BacA is essential for the chronic infection of nodules as well as bacteroid development (12, 14).

We investigated whether the BCR peptides of *A. pisum* exhibit antimicrobial activity and affect cell membrane permeability, similar to the NCR peptides of legume plants. We treated *E. coli*, a model γ-proteobacterium closely related to *Buchnera*, with chemically synthesized BCRs. We found that 4 out of the 6 BCR peptides assayed exhibited antimicrobial activities and induced cell elongation and higher intensities of 4′,6-diamidino-2-phenylindole (DAPI) and propidium iodide (PI) fluorescence. The *E. coli sbmA* mutant, a homolog of *bacA* of *S. meliloti*, was more sensitive to BCR peptides than the wild type. Similarities in the *in vitro* activities of BCR peptides to those of NCR peptides (14, 34) suggest that BCR peptides are involved in the symbiosis with *Buchnera* in the pea aphid in a similar manner to NCR peptides in legume plants.

## Materials and Methods

### Bacterial strains and media

*E. coli* wild-type strains MG1655 and BW25113 and the *sbmA*-disrupted mutant JW0368 derived from BW25113 (1) were provided by the National BioResource Project (https://shigen.nig.ac.jp/ecoli/strain) and maintained on Luria-Bertani (LB) medium. *S. meliloti* 1021 and its *bacA* mutant (8) were maintained on TY medium (3). In bioassays of the activities of BCR and NCR peptides, all strains were cultured in M9 liquid medium (25) supplemented with 0.2% glucose. In estimations of colony-forming units (cfus), *E. coli* strains were plated on LB agar plates and *Sinorhizobium* strains on TY agar plates.

### Refolding of BCR peptides

BCR1, BCR2, BCR4, BCR5, and BCR8 peptides were chemically synthesized through a custom peptide synthesis service by Medical & Biological Laboratories (Nagoya, Japan) and BCR3 was synthesized by Biomatik Corporation (Cambridge, Canada). They were refolded with a Refolding CA Kit (Takara Bio, Kusatsu, Japan) according to the manufacturer’s instructions. In brief, peptides were unfolded using guanidine hydrochloride with dithiothreitol, and were refolded in cycloamylose/Tween 40 with D,L-cystine. They were then passed through an Oasis HLB 3 cc column (Nihon Waters, Tokyo, Japan) and eluted with 1 mL of elution buffer (4 vol. acetonitrile : 1 vol. methanol : 5 vol. Milli-Q water containing 0.1% trifluoroacetic acid). Eluates were dried and then dissolved in Milli-Q water to a final concentration of 2 mg mL^−1^. The refolded peptide was verified by HPLC, and the formation of disulfide bonds was confirmed by mass spectrometry. BCR6 was not assayed in the present study because BCR6 is too long (84 aa) to synthesize chemically. The NCR247 peptide was synthesized and used without refolding.

### Treatment of bacterial strains with BCR or NCR peptides

All bacterial strains were cultured in liquid M9 medium. When the OD_600_ of the culture reached 0.3, cells were harvested and washed with 10 mM Tris·HCl buffer (pH 7.5) three times. Cells were then suspended in 10 mM Tris·HCl buffer (pH 7.5) to OD_600_=0.1. Each test peptide was added to the bacterial suspension at an appropriate concentration and the suspension was incubated at 30°C for 3 h. In the analysis of cell morphology and membrane permeability, BCR peptides were added to a final concentration of 5 μM. As a control treatment, bovine serum albumin (BSA) was used at the same concentration as BCR peptides.

### Detection of antimicrobial activities of BCR and NCR peptides

To estimate antimicrobial activities, we diluted the bacterial suspension treated with each peptide and spread it on LB (*E. coli*) or TY (*S. meliloti*) agar plates. The relative number of cfus was assessed in relation to the number that appeared on control (BSA-treated) plates as 100%. To investigate changes in morphology and membrane permeability, we analyzed the bacterial suspension according to previous studies (15, 34) that analyzed the activities of NCR peptides. Bacterial cells are detectable by staining with the fluorescent DNA dye DAPI. PI, a fluorescent dye that stains nucleic acids, is excluded from living cells, but enters dead cells or cells with the loss of membrane integrity. In brief, we added DAPI and PI together to the suspension to a final concentration of 10 μg mL^−1^ each and analyzed cells using a cell sorter (SH800, Sony, Tokyo, Japan) and confocal microscopy (A1, Nikon Instech, Tokyo, Japan). The cfus of *E. coli* suspensions and morphology and membrane permeability of *E. coli* cells were not affected by BSA under the experimental conditions employed in the present study (data not shown).

## Results

### Antimicrobial activities of synthetic BCR peptides

**Fig. 1:**
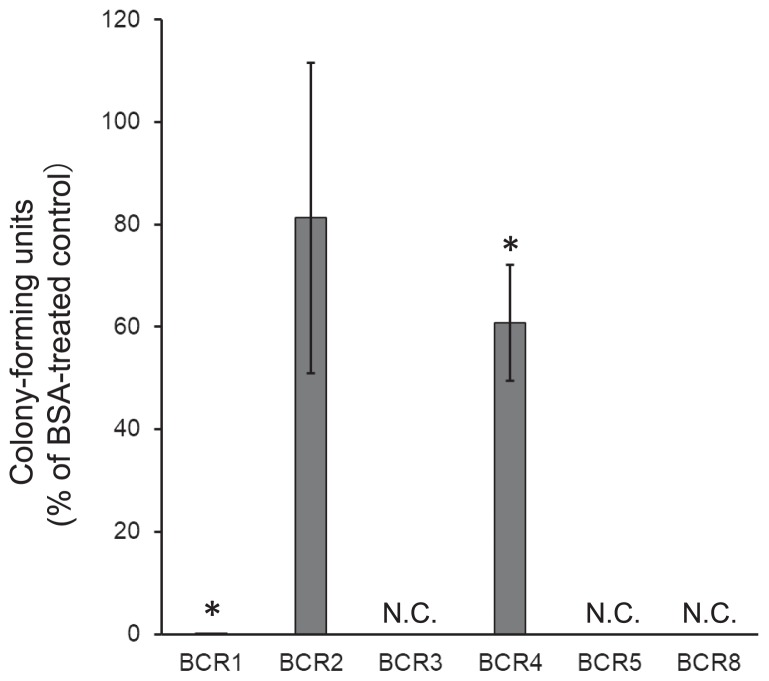
Antimicrobial activities of BCR peptides. *E. coli* MG1655 was treated with 5 μM BCR peptides for 3 h and colony-forming units were estimated relative to the BSA control. Each value is the mean±SE of three independent experiments. Asterisks indicate a significant difference between BCR4 and the control (*P*<0.05 by the Student’s *t*-test). N.C., no colony appeared.

BCR1, BCR3, BCR5, and BCR8 at 5 μM exhibited strong antimicrobial activities against *E. coli* (MG1655) cells; the latter three prevented colony formation (Fig. 1). When *ca*. 5×10^7^ cells were treated with 5 μM of BCR1, *ca*. 1×10^2^ cells survived (data not shown). No colony formed from the bacterial suspension treated with BCR3, BCR5, or BCR8. BCR4 exhibited mild antimicrobial activity, whereas BCR2 showed no significant antimicrobial activity (Fig. 1).

### Effects of BCR peptides on *E. coli* cells

**Fig. 2:**
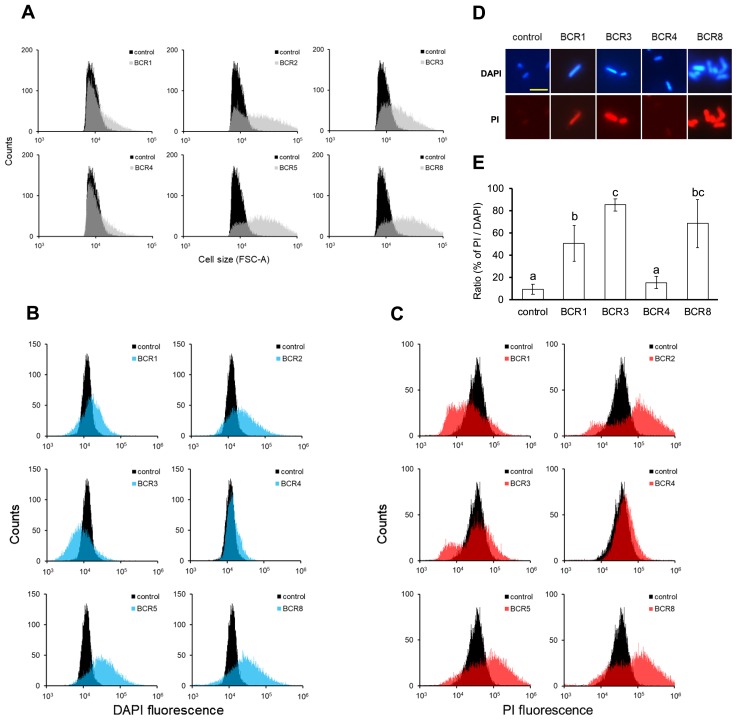
Effects of BCR peptides on *E. coli* cells. *E. coli* MG1655 cells were treated for 3 h with 5 μM of the BCR peptide or BSA as the control. Cells were stained with DAPI and PI and analyzed by a cell sorter. (A) Forward scatter (FSC), (B) DAPI, (C) PI. (D) Fluorescent microscopy of MG1655 cells treated with BCR peptides and stained with DAPI and PI. Images are representative micrographs of cells treated with each peptide. Scale bars, 5 μm. (E) Ratio of PI-positive cells to DAPI-stained cells. At least 4,000 cells were counted in each treatment. Each value is the mean±SE. Means denoted by the same letter do not differ significantly (*P*<0.05, the Student’s *t*-test).

To reveal the effects of BCR peptides on cell morphology, we used the cell sorter to measure the forward scatter parameter (FSC), which indicates cell size, and the side scatter parameter (SSC), which indicates the complexity of granularity and internal complexity of cells, including cell formation. Histograms of FSC and SSC measured by the cell sorter are shown in Fig. 2A. BCR2, BCR3, BCR5, and BCR8 increased FSC (Fig. 2A) and SSC (Fig. S1). BCR1 and BCR4 slightly increased FSC (Fig. 2A), but not SSC (Fig. S1).

Most control cells stained with DAPI fluoresced at intensities of 20,000–30,000 (Fig. 2B). BCR1-treated and BCR3-treated cells produced a wider range of the signal than control cells, and BCR1-treated cells increased the frequency at higher intensities (Fig. 2B). Cells treated with BCR2, BCR5, and BCR8 fluoresced at higher intensities (Fig. 2B). BCR4-treated cells produced slightly higher intensities (Fig. 2B). BCR1, BCR2, BCR3, BCR5, and BCR8 clearly changed the profile of PI staining, shifting it to higher intensities and a wider distribution (Fig. 2C). BCR1 widened the distribution, shifting the main frequency to a lower intensity and slightly increasing the higher intensities (Fig. 2C). BCR4 slightly shifted the distribution to a higher intensity (Fig. 2C). In summary, cell sorter analyses revealed that some of the BCRs affected cell morphology and membrane permeability, which increased the intensities of DAPI and PI staining.

BCR-treated *E. coli* cells were also inspected by fluorescence microscopy. BCR1 and BCR3 significantly elongated cells and increased the intensities of DAPI and PI fluorescence (Fig. 2D). BCR3 and BCR8 promoted cell aggregation (Fig. 2A, 2D, and S1). BCR4 had no significant effect on either morphology or fluorescence. BCR1, BCR3, and BCR8 significantly increased the fraction of PI-positive cells (Fig. 2E).

### Sensitivity of the *sbmA* mutant to BCR peptides and NCR247

**Fig. 3:**
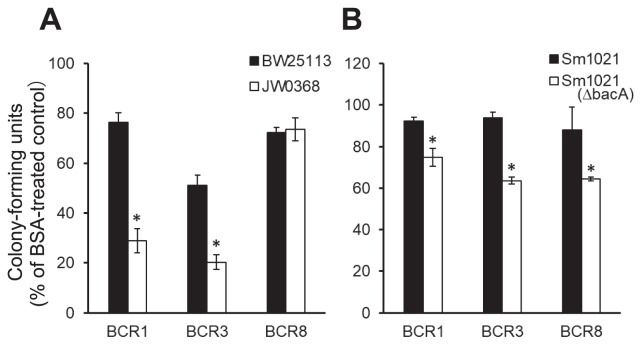
Sensitivity of the *E. coli sbmA* mutant and *S. meliloti* Δ*bacA* mutant to BCR peptides. After a treatment with 3 μM BCRs for 3 h, the colony-forming units of each strain were estimated relative to the BSA control. The dataset shown is representative of three independent experiments. (A) *E. coli* BW25113 (wild type) and JW0368 (*sbmA* mutant). (B) *S. meliloti* 1021 and Δ*bacA mutant*. Each value is the mean±SE of three independent experiments. Asterisks indicate a significant difference between the wild type and mutant (*P*<0.01, the Student’s *t*-test).

To reveal whether the SbmA protein is related to the anti-microbial activity of BCR peptides, BCR1, BCR3, and BCR8 were used because these BCRs exhibited strong antimicrobial activity. We treated two strains of *E. coli*, BW25113 (wild type) and JW0368 (*sbmA* mutant), with the BCR peptides at lower concentrations and calculated cfu values. Since no colonies formed when BW25113 and JW0368 were treated with 5 μM BCR1, BCR3, or BCR8 (Fig. S2), we used a sub-lethal concentration, 3 μM (Fig. 3A), in subsequent assays. BCR1 and BCR3 reduced the cfus of JW0368 to less than those of BW25113 (Fig. 3A). BCR1, BCR3, and BCR8 also caused the elongation of BW25113 and JW0368 cells (Fig. S3).

*S. meliloti* Δ*bacA* was significantly more sensitive to 20 μM NCR247 than wild-type *S. meliloti* 1021 (Fig. S4), as previously reported (14). JW0368, an *E. coli sbmA* mutant, was more sensitive to NCR247 at 5 μM, but not at 20 μM, than BW25113 (Fig. S4). Both *E. coli* strains were more resistant to NCR247 than *S. meliloti* (Fig. S4). No *S. meliloti* colonies formed at 5 μM BCR1, BCR3, or BCR8 (Fig. S5). Strain 1021 was resistant to 3 μM BCR1, BCR3, and BCR8, whereas the Δ*bacA* mutant was sensitive to them (Fig. 3B).

**Fig. 4:**
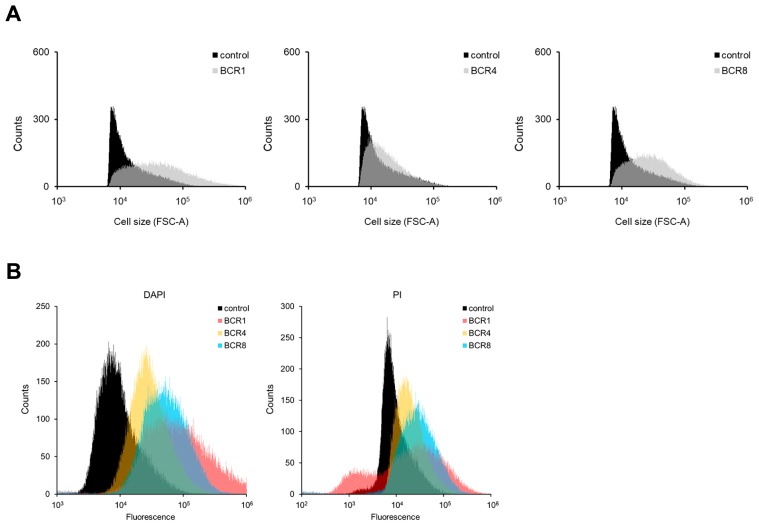
Effects of BCR peptides on *S. meliloti*. *S. meliloti* 1021 cells were treated for 3 h with 5 μM BCR peptides or 5 μM BSA. Cells stained with DAPI and PI were analyzed by flow cytometry. (A) Forward scatter (FSC), (B) DAPI and PI. Data are representative of at least three independent experiments.

The effects of BCR1 and BCR8 on wild-type *S. meliloti* cells was analyzed using the cell sorter. BCR1 and BCR8 markedly increased FSC (Fig. 4A), SSC (Fig. S6), DAPI, and PI fluorescence (Fig. 4B). Thus, BCR1 and BCR8 exerted similar effects on *S. meliloti* as those on *E. coli*. BCR4 showed similar, but weaker effects.

The observed effects of BCR/NCR peptides on both *E. coli* and *S. meliloti* are summarized in Supplementary Tables S1 and S2.

## Discussion

We investigated the antimicrobial activities of synthesized BCR peptides found in the bacteriocytes of pea aphids. Seven genes for BCR peptides (BCR1–6, 8) have been identified in the genome of *A. pisum* (31). The mRNA expression of BCRs in the embryo is initiated around the developmental stage coincident with the infection of *Buchnera* into the embryo from maternal bacteriocytes, and mRNA expression is maintained exclusively in bacteriocytes throughout the life of the aphid (31). Although expression patterns strongly suggest that BCR peptides control endosymbionts, their functions and activities have not yet been investigated. We synthesized 6 out of 7 *A. pisum* BCR peptides and examined their effects on bacteria. All peptides exhibited antimicrobial activity or permeabilized the membrane of *E. coli* cells: BCR1, BCR3, BCR5, and BCR8 exhibited strong antimicrobial activity and permeabilized the cell membrane, BCR2 only increased permeabilization, and BCR4 only showed mild antimicrobial activity (Fig. 1 and 2). Thus, at least four aphid BCR peptides were identified as antimicrobial peptides (AMPs). There was no obvious correlation detectable among the amino acid sequences, isoelectric points, and antimicrobial activities of BCR peptides. Since the sequence database search of BCR peptides returned no significant hits outside of aphid species (31), these BCR peptides constitute a novel class of AMPs specific to the aphid lineage.

We found that some BCRs exerted antimicrobial effects, whereas other did not. We also noted that each BCR peptide exerted antimicrobial effects at a different level and their effects on bacterial morphology and membrane permeability varied. In *M. truncatula*, the genes for NCR peptides are expressed exclusively in the root nodules, whereas expression patterns differ among genes (22). Each NCR peptide localizes to a different zone of the nodule; *e.g*., NCR035 localizes to the interzone and nitrogen-fixing zone, while NCR001 only localizes to the nitrogen-fixing zone (34). NCR035 and NCR247 exhibit strong antimicrobial activities against *S. meliloti*, while some NCR peptides, such as NCR057 or NCR224, do not. The genes for all six BCR peptides used in the present study are exclusively expressed in the bacteriocyte of the pea aphid (31). Among them, BCR2 and BCR4, similar to NCR057 and NCR224, did not exhibit strong antimicrobial activities; BCR2 affected cell morphology and membrane permeability without antimicrobial activity. These results suggest that each BCR plays a different role in symbiosis, similar to NCR peptides. Although the location and target of the peptides within the bacteriocyte have not yet been reported, the pea aphid also has a diverse line-up of CRPs that may function in a different context in symbiosis, as observed in *M. truncatula*.

Legumes, such as *M. truncatula*, of the Inverted-Repeat-Lacking Clade (IRLC) use several hundred NCR peptides to control their microsymbionts. *Aeschynomene* spp. legumes, of the more ancient dalbergoid lineage, are expected to have several tens to hundreds of NCR-like peptides (7). On the other hand, only seven genes for BCR peptides have been identified on the genome of *A. pisum*. Although the reason for the marked difference in the number of CRPs between aphid–*Buchnera* and legume–rhizobia symbioses remains unclear, different systems of symbiosis may be responsible: Rhizobia are soil bacteria that may survive independently of their host plant; therefore, during symbiosis, the host plant may need to tightly control them by using NCR peptides with diverse functions. In contrast, *Buchnera* has lost the ability to survive outside of the pea aphid and, thus, may be controlled by BCR peptides with very restricted functions. Further studies are required to understand the diverse evolutionary processes of CRPs among symbiotic systems.

BacA, a membrane protein of *S. meliloti*, is essential for symbiosis with *M. sativa* (12). BacA of *S. meliloti* is involved in the modification of lipids with fatty acids (9); however, the molecular mechanisms employed by the BacA protein for resistance to AMPs remain unknown. The *bacA* mutant of *S. meliloti* is sensitive to NCR peptides and is unable to differentiate into bacteroids in host nodule cells, resulting in the abortion of symbiosis (14). *SbmA* of *E. coli* is a homolog of *BacA* of *S. meliloti* and may complement the symbiosis defect of the *S. meliloti bacA* mutant (16). Although differences were small, the significantly greater sensitivity of the *E. coli sbmA* mutant and *S. meliloti bacA* mutant to BCR1, BCR3, and NCR247 than their parent strains (Fig. 3 and S4) suggests the involvement of bacterial SbmA and BacA proteins in sensitivity to these cysteine-rich AMPs and the similar function of these BCR peptides to NCR peptides. BacA and SbmA are both transporters that import a number of structurally diverse peptides into the cell (11, 18, 20, 21, 36). In *S. meliloti*, BacA is essential for bacteroid differentiation and survival in the host plant cell (12, 14). However, we did not find any homologs of *bacA/sbmA* in the *Buchnera* genome. BCR peptides may function via a BacA/SbmA-independent mechanism and affect the membrane permeability and survival of *Buchnera*.

AMPs are common peptides that function in the innate immunity of eukaryotes (37). Besides BCR peptides and NCR peptides, AMPs that are expressed and/or function in symbiotic organs have been reported in other symbiotic relationships: *e.g*., between bean bugs and *Burkholderia* (10) and between actinorhizal plants and *Frankia* (6). The weevil antimicrobial coleoptericin-A peptide regulates the growth of its symbiont by inhibiting cell division (19). In the symbiosis between the actinorhizal plant *Alnus* and *Frankia*, *Alnus* provides defensin-like peptide Ag5 to *Frankia*. The present study revealed that some BCR peptides exhibited antibiotic activity and increased the permeabilities of *E. coli* and *S. meliloti* cells. A possible function of antimicrobial BCR peptides is to control the growth of *Buchnera* in bacteriocytes, similar to coleoptericin-A, Ag5, and NCR, thereby controlling the size of the symbiont population within the host. These antimicrobial BCRs may also interact with secondary symbionts or invading microbes in the aphids. Another possible function of BCRs is to promote metabolite exchange (24). *In vitro*, Ag5 exhibits antimicrobial activity against *Frankia* and, at sublethal concentrations, induces the permeabilization of the vesicle membrane, resulting in the release of amino acids, particularly glutamine and glutamate, from *Frankia* cells (6, 24), which contributes to metabolic exchange between *Frankia* and nodule cells. Since the pea aphid has lost many transporter genes in its genome (24, 29), BCR peptides that permeabilize the cell membrane may be used in the exchange of metabolites between *Buchnera* and host cells. Animals and plants may both use AMPs not only as a defense against microbial attack, but also for symbiosis with microbes, representing a parallel evolution in symbiosis.

## Supplementary Information
