# Supplementary material for: Antimicrobial Activities of Cysteine-rich Peptides Specific to Bacteriocytes of the Pea Aphid Acyrthosiphon pisum
Source: Microbes Environ. 2019 Mar 21;34(2):155–60. doi: 10.1264/jsme2.ME18148 (PMC6594739; doi:10.1264/jsme2.ME18148)
Supplement: Supplementary file 1 [file 34_155_s1.pdf]

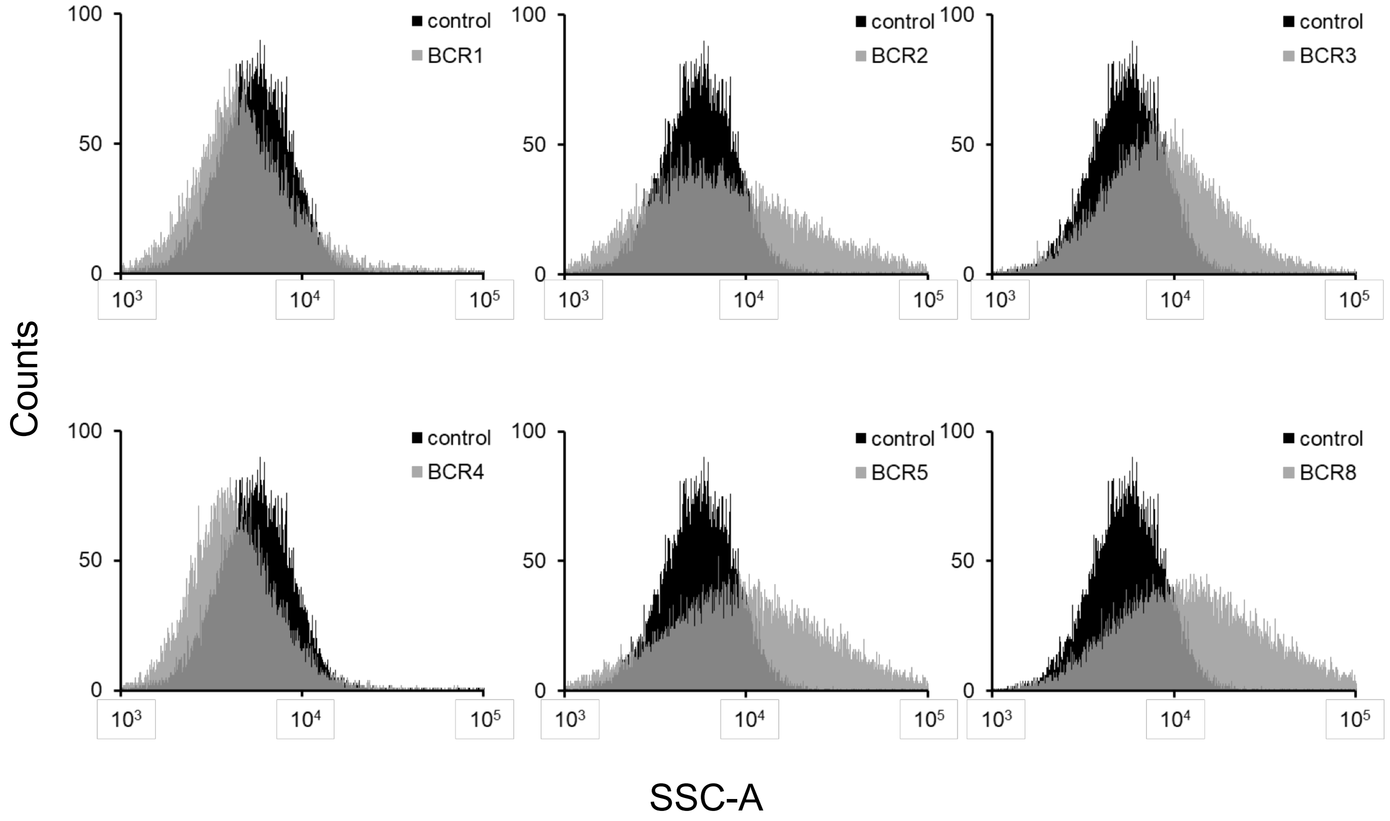

Fig. S1. Effects of BCR peptides on *E. coli* cells. *E. coli* MG1655 cells were treated for 3 h with 5 μM BCR peptides or BSA as a control. Cells were stained with DAPI and PI, and analyzed by cell sorter. Histograms show side scatter (SSC).

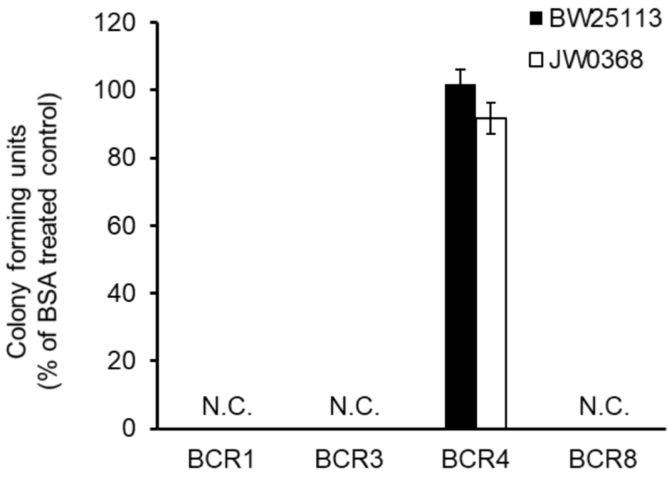

Fig. S2. Sensitivity of *E. coli sbmA* mutant to BCR peptides. After treatment with 5 μM BCRs for 3 h, colony-forming units of *E. coli* BW25113 (wild type) and JW0368 (*sbmA* mutant) were estimated relative to BSA control. Each value is the mean ± SE of three independent experiments. There were no significant difference between wild type and *bacA* mutant (P < 0.05, Student's *t*-test).

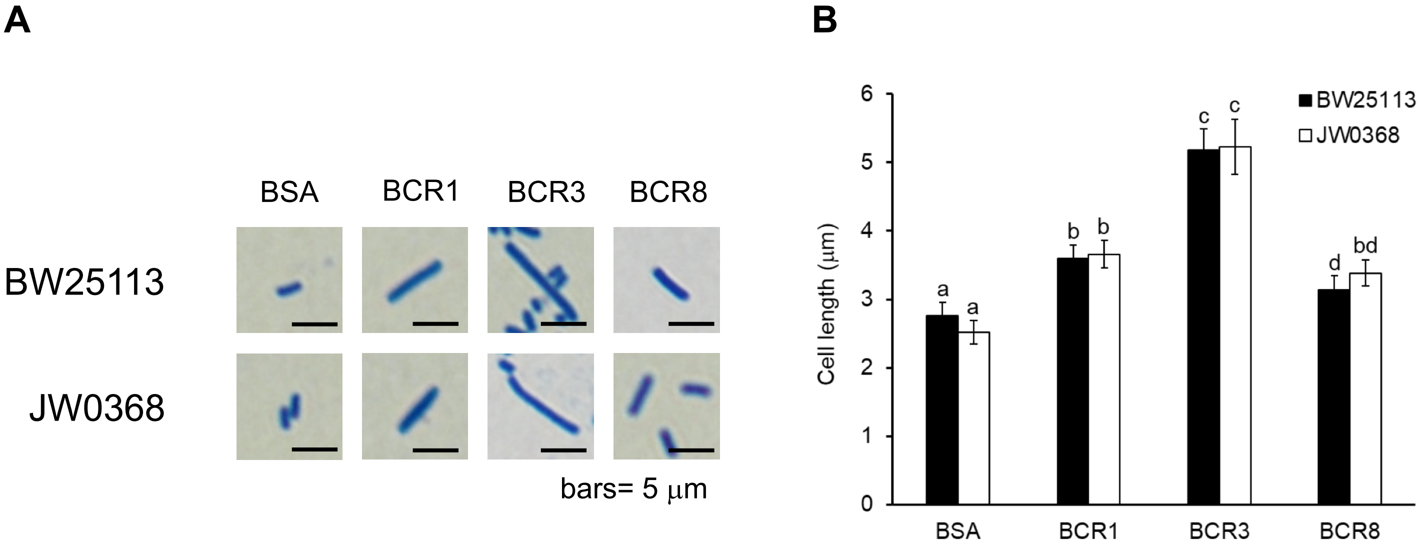

Fig. S3. (A)The cells of BW25113 and JW0368 were treated with 5 μM BCR peptides, fixed with methanol (Magee *et al*., 1975) and stained with toluidine blue for 30 min. Images are representative micrographs of cells treated with each peptide. Scale bars, 5 μm. (B)The cell lengths of BW25113 and JW0363 were treated with BCR peptides. Means denoted by the same letter do not differ significantly (P < 0.05, Student's *t*-test).

Magee, C. M. *et al*., 1975. A more reliable gram staining technic for diagnosis of surgical injections. Am. J. Surg. 130:341-346

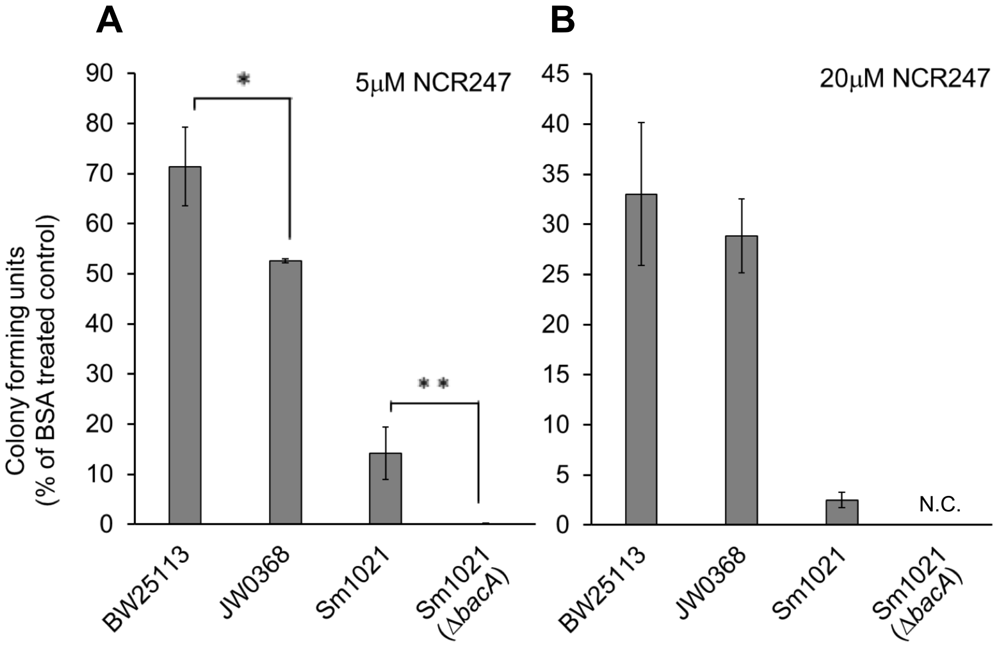

Fig. S4. Antimicrobiotic activity of NCR247 against *E. coli* and *S. meliloti*. After treatment with (A) 5 μM or (B) 20 μM NCR247 for 3 h, colony-forming units of each strain were estimated relative to BSA control. The dataset shown is representative of trends observed in three independent experiments. Each value is the mean ± SE of three independent experiments. Asterisks indicate significant difference between NCR247 treated cells and control (*, $P < 0.05$ by Student's *t*-test; **, $P < 0.001$ by Student's *t*-test). N.C., no colony appeared.

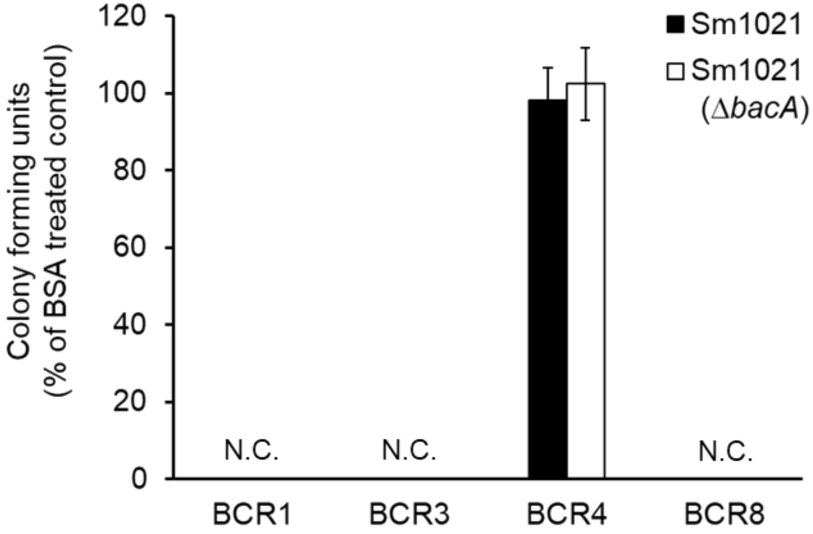

Fig. S5. Sensitivity of *S. meliloti bacA* mutant to BCR peptides. After treatment with 5 μM BCRs for 3 h, colony-forming units of *S. meliloti* 1021 (wild type) and the *bacA* mutant were estimated relative to BSA control. Each value is the mean ± SE of three independent experiments. There were no significant difference between wild type and *bacA* mutant ($P < 0.05$, Student's *t*-test).

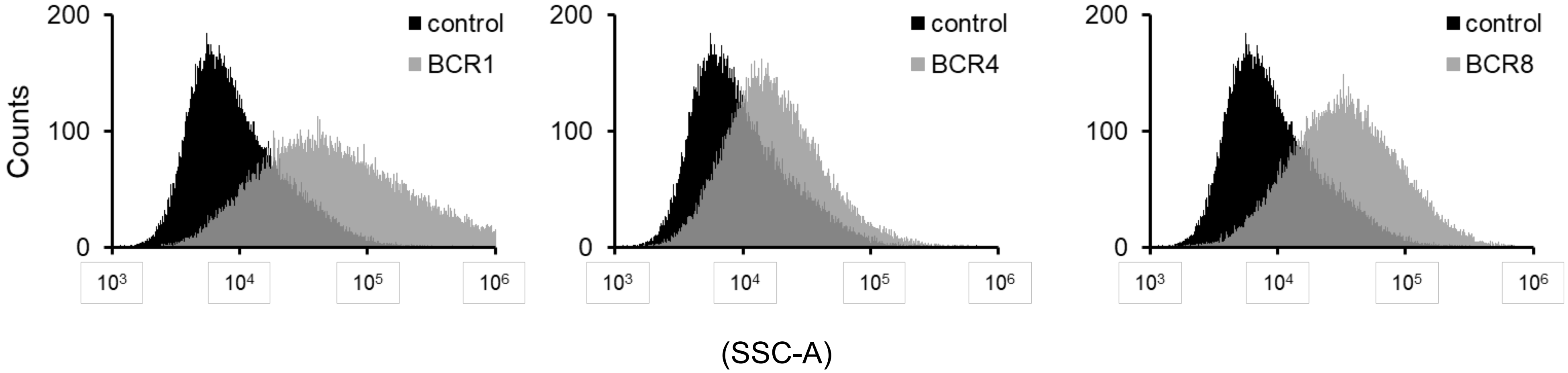

Fig. S6. Effects of BCR peptides on *S. meliloti* 1021. *Sinorhizobium meliloti* 1021 cells were treated for 3 h with 5 μM BCR peptide or BSA as a control. Cells were stained with DAPI and PI, and analyzed by cell sorter. Histograms show side scatter (SSC) respectively.

Table S1. Activities of BCR peptides or NCR247 on *E. coli* and *S. meliloti* cells.

	test	BCR1	BCR2	BCR3	BCR4	BCR5	BCR8	NCR247
E. coli	Antibacterial activity	+	-	+	±	+	+	+
	Cell length	±	+	+	-	+	+	+
	DAPI Fluorescence	+	+	+	-	+	+	+
	PI Fluorescence	+	+	+	-	+	+	+
S. meliloti	Antibacterial activity	+	n.d.	+	-	n.d.	+	+[1]
	Cell length	+	n.d.	n.d.	±	n.d.	+	+[2]
	DAPI Fluorescence	+	n.d.	n.d.	±	n.d.	+	n.d.
	PI Fluorescence	+	n.d.	n.d.	±	n.d.	+	+[2]

+, obvious change; ±, slight change; −, not detectable. n.d., no data.

References

1. Haag *et al*., 2012. Role of cysteine residues and disulfide bonds in the activity of a legume root nodule-specific, cysteine-rich peptide. J. Biol. Chem. 287:10791-10798
2. Van de Velde *et al*. 2010. Plant peptides govern terminal differentiation of bacteria in symbiosis. Science. 327:1122–1126

Table S2. Activities of BCR peptides or NCR247 on *sbmA/bacA* mutant cells compared with the wild-type strains.

	test	BCR1	BCR3	BCR4	BCR8	NCR247
E. coli JW0368 (*sbmA* mutant)	Antibacterial activity	+	+	-	-	+
	Cell length	-	-	-	-	-
S. meliloti Sm1021 Δ*bacA*	Antibacterial activity	+	+	-	+	+
	Cell length	-	-	-	-	-

+, more sensitive; -, no significant difference compared with the wild-type. Each strain was treated with 3 μM of BCR1, BCR3, BCR8 or 5 μM of BCR4, or 5 μM of NCR247.
